# Differential relationships of PTSD symptom clusters with cortical thickness and grey matter volumes among women with PTSD

**DOI:** 10.1038/s41598-020-80776-2

**Published:** 2021-01-19

**Authors:** Kevin M. Crombie, Marisa C. Ross, Allison M. Letkiewicz, Anneliis Sartin-Tarm, Josh M. Cisler

**Affiliations:** Department of Psychiatry, University of WI – Madison, 6001 Research Park Boulevard, Madison, WI 53719-1176 USA

**Keywords:** Neuroscience, Psychology

## Abstract

Structural neuroimaging studies of posttraumatic stress disorder (PTSD) have typically reported reduced cortical thickness (CT) and gray matter volume (GMV) in subcortical structures and networks involved in memory retrieval, emotional processing and regulation, and fear acquisition and extinction. Although PTSD is more common in women, and interpersonal violence (IPV) exposure is a more potent risk factor for developing PTSD relative to other forms of trauma, most of the existing literature examined combat-exposed men with PTSD. Vertex-wise CT and subcortical GMV analyses were conducted to examine potential differences in a large, well-characterized sample of women with PTSD stemming from IPV-exposure (n = 99) compared to healthy trauma-free women without a diagnosis of PTSD (n = 22). Subgroup analyses were also conducted to determine whether symptom severity within specific PTSD symptom clusters (e.g., re-experiencing, active avoidance, hyperarousal) predict CT and GMV after controlling for comorbid depression and anxiety. Results indicated that a diagnosis of PTSD in women with IPV-exposure did not significantly predict differences in CT across the cortex or GMV in the amygdala or hippocampus compared to healthy controls. However, within the PTSD group, greater re-experiencing symptom severity was associated with decreased CT in the left inferior and middle temporal gyrus, and decreased CT in the right parahippocampal and medial temporal gyrus. In contrast, greater active avoidance symptom severity was associated with greater CT in the left lateral fissure, postcentral gyrus, and middle/lateral occipital cortex, and greater CT in the right paracentral, posterior cingulate, and superior occipital gyrus. In terms of GMV, greater hyperarousal symptom severity was associated with reduced left amygdala GMV, while greater active avoidance symptom severity was associated with greater right amygdala GMV. These findings suggest that structural brain alterations among women with IPV-related PTSD may be driven by symptom severity within specific symptom clusters and that PTSD symptom clusters may have a differential (increased or decreased) association with brain structures.

## Introduction

Posttraumatic stress disorder (PTSD) is a highly debilitating mental health disorder characterized by intrusive recollection of traumatic memories, avoidance of people and places that trigger recall of traumatic memories, and prolonged physiological hyperarousal and vigilance towards danger^1^. Although individuals can develop PTSD following exposure to a broad range of traumatic events (e.g., combat, motor vehicle accidents), epidemiological evidence suggests that interpersonal violence (IPV) exposure (i.e., physical or sexual assault) is a more potent risk factor than other forms of trauma^2–4^. PTSD, which is more common in women than men^5–7^, is associated with markedly elevated morbidity, distress, and decreased quality of life relative to other anxiety-related disorders^8^. Psychotherapeutic approaches (e.g., exposure-based therapy) are among the best supported interventions for PTSD, yet remission rates for these front-line treatments are typically only 50–60%^9,10^. Thus, a better mechanistic understanding of PTSD across various levels (e.g., neural, molecular, cellular) and modalities (e.g., neuroimaging) may contribute to the development or enhancement of novel or existing therapies, or potentially elucidate why certain therapies have yielded sub-optimal efficacy rates.

Advances in functional and structural neuroimaging techniques and computational analytic approaches has led to an improved understanding of neural correlates of PTSD. Much of our understanding of the structural correlates of PTSD stems from studies examining subcortical gray matter volume (GMV) and structural alterations in cortical thickness (CT) among combat-exposed men with PTSD. For instance, Bremner et al. provided the first evidence of a significant reduction in hippocampal GMV of patients with combat related PTSD compared to age and sex-matched healthy controls^11^, a finding that has since been replicated in this population^12–15^. Since this seminal study, numerous independent investigations and meta-analytic evidence largely supports the notion that a diagnosis of PTSD stemming from several sources of trauma (e.g., combat-related trauma, motor vehicle accidents, natural disaster survivors) is associated with reduced GMV in subcortical structures and reduced CT in several regions, including but not limited to the amygdala, prefrontal cortex (medial, ventral, dorsolateral), inferior, superior, postcentral, and middle temporal gyri, and anterior and posterior cingulate cortex^16–27^. Accordingly, deficits in CT and subcortical volumes within this population are present in regions and networks involved in memory retrieval, emotional processing and regulation, decision-making, and fear acquisition and extinction, all processes known to be altered in PTSD^28–30^.

In contrast, there is a scarcity of investigations examining potential differences in GMV and CT in adult women with PTSD specifically resulting from IPV-exposure, although there is a recent increase in research examining women with PTSD in general^31,32^. The lack of research within this population is unfortunate as: (1) women are more than twice as likely to develop a diagnosis of PTSD following trauma exposure^5–7^, and (2) the probability of developing PTSD is greatest following IPV exposure, which is more common in women^4,7,33–35^. Given known sex differences in PTSD in prevalence, exposure to physical and sexual assault, and comorbid depression and anxiety (all with greater rates in women), it is important to probe structural correlates among women with PTSD specifically^4–7,33–36^. One of the few investigations conducted to date examined a small sample of women experiencing chronic PTSD (n = 17) following sexual abuse and found no difference in global and regional brain volumes and CT compared to age and education-matched controls^37^. Relatedly, an investigation of women (n = 11) with interpersonal partner violence histories^38^ found no evidence of gray matter deficits within subcortical structures (e.g., hippocampus), which has been commonly reported in male samples with combat related PTSD^11,13–15^. In contrast, a small sample (n = 15) consisting primarily of women with PTSD stemming from non-combat related traumatic experiences (66% of sample consisted of women, 87% of sample experienced IPV) reported a positive relationship between avoidance symptoms and right amygdala and hippocampal volume^39^. Additionally, literature examining childhood trauma (which incorporates interpersonal trauma) and CT has yielded equivocal findings as a recent report^40^ indicated that adolescents with childhood sexual abuse (85% of sample consisted of females) preserved CT (in all a priori regions of interest), while other investigations (consisting of both adolescents and adult males and females) have reported altered CT throughout the brain^41–43^. Overall, our understanding of potential differences in GMV and CT in adult women with PTSD resulting from IPV-exposure could benefit from investigations with larger sample sizes.

Another important consideration that has not been thoroughly examined within this population is differentiating structural correlates of PTSD from depression and general anxiety. Meta-analytic evidence indicates that depressed individuals often exhibit structural differences (e.g., decreased GMV within subcortical limbic brain regions) similar to those commonly reported among individuals with PTSD^44–46^. Given that depression and anxiety are highly comorbid in women with IPV-related PTSD^7,47–49^, it is important for investigations to also include and control for depression and anxiety in structural analyses, so that we are better able to identify the unique contribution of PTSD. Relatedly, previous investigations have primarily focused on overall PTSD symptom severity, while neglecting to examine whether symptom severity within specific PTSD symptom clusters predicts different structural correlates. Examining such relationships could prove important as differences in symptom severity within clusters has previously been found to differentially influence interpersonal functioning, physical health, and comorbid mental health diagnoses, including depression^50,51^.

Given the differential structural findings (i.e., a potential lack of structural differences in women with IPV-exposure) compared to primarily male combat veteran populations, the limited existing data highlight the importance of further examining the neural mechanisms of physical and sexual assault-related PTSD in larger samples of women, for which the traumatic experience is vastly different. Furthermore, the association between CT, GMV, and PTSD symptom severity (for each symptom cluster) within a sample of women with IPV-related PTSD, to our knowledge, has not yet been investigated. As a result, the current study sought to conduct vertex-wise CT and subcortical GMV analyses to examine potential differences in a large, sample of adult women with PTSD stemming from IPV-exposure (n = 99) compared to non-trauma exposed adult women without a diagnosis of PTSD (n = 22). Given the substantial symptom heterogeneity in PTSD, this study also sought to move beyond a simple dichotomized comparison (PTSD vs. no PTSD) by conducting subgroup analyses to determine whether symptom severity within specific PTSD symptom clusters (e.g., re-experiencing, avoidance, hyperarousal) predicts CT and GMV in adult women with PTSD from IPV-exposure. Based on prior investigations (primarily examining men) and meta-analytic evidence^11–27,39^, it was hypothesized that (1) women with PTSD stemming from IPV-exposure would exhibit reduced amygdala and hippocampal GMV, and reduced CT in several regions, including: prefrontal cortex (medial, ventral, dorsolateral), inferior, superior, postcentral, and middle temporal gyri, and anterior and posterior cingulate cortex compared to non-trauma exposed women without a diagnosis of PTSD; and (2) among women with PTSD, greater avoidance symptom severity scores would predict greater right amygdala and hippocampal volume.

## Materials and methods

The work described in this manuscript has been carried out in accordance with The Code of Ethics of the World Medical Association (Declaration of Helsinki) for experiments involving humans, and all subjects completed informed consent.

### Participant recruitment

Adult females aged 21–50 were recruited through three different studies conducted at two sites: the University of Arkansas for Medical Sciences (UAMS) and the University of Wisconsin—Madison (UW). All study procedures were approved by the Institutional Review Board at UAMS and the UW Health Sciences Institutional Review Board, and all methods were carried out in accordance with relevant guidelines and regulations. The analyses included in the current manuscript are independent of previously reported findings^52–55^. These studies recruited women with a primary diagnosis of PTSD and a primary index trauma of IPV-exposure, along with healthy women, free of trauma exposure or mental health concerns. Inclusion criteria for the PTSD group in all studies was a primary diagnosis of PTSD, IPV-exposure, and female sex. Inclusion criteria for the healthy control groups included female sex, absence of trauma exposure, absence of current psychiatric disorders, and absence of psychiatric medication use. Exclusion criteria for all participants included internal metal or other MRI contraindications, major medical disorders, and endorsement of psychotic symptoms.

### Assessments

Participants completed a baseline demographic questionnaire assessing age, race, and education. Trauma histories were assessed with the National Women’s Survey (NSA)^56^ trauma assessment section, which is a structured interview that assesses previous history of physical abuse by a caregiver, physical assault, sexual assault, witnessed domestic violence, witnessed community violence, and a range of other stressful life events. The presence of mental health and psychiatric disorders (e.g., major depressive disorder, anxiety disorders) was assessed using the Structured Clinical Interview for DSM-IV^57^. PTSD diagnosis and symptom severity was assessed using the Clinician Administered PTSD Scale for DSM-5 (CAPS-5)^58^ and the PTSD Checklist for DSM-5 (PCL-5)^59^ for two studies (n = 110) and the Structured Clinical Interview for DSM-IV Disorders (SCID-IV) and PTSD Checklist-Civilian Version (PCL-C)^60^ for one study (n = 30). Of the 121 participants included in the analyses, 11 participants (50%) from the control group and 15 participants (15%) from the PTSD group completed DSM-IV measures, while 11 participants (50%) from the control group and 84 participants (85%) from the PTSD group completed DSM-V measures. PCL-5 scores were used as the measure of PTSD symptom severity for each cluster and for overall symptom severity in all analyses including PTSD symptoms. As a result, PCL-C scores were converted to PCL-5 scores using an established and validated crosswalk procedure using equipercentile equating^61^. All interview-based assessments were conducted by trained clinical interviewers. See supplementary material for correlation table of assessment factors.

### Data acquisition

At the UAMS site (n = 72), T1-weighted anatomic images were acquired with an MP-RAGE sequence (matrix = 192 × 192, 160 sagittal slices, TR/TE/FA = 7.5/3.7/9°, FOV = 256, 256, 160, final resolution = 1 × 1x1 mm) on a Philips Achieva 3 T X-Series scanner with a 32-channel headcoil. At the UW site (n = 68), T1-weighted anatomic images were acquired with a similar MP-RAGE sequence (matrix = 256 × 256, 156 axial slices, TR/TE/FA = 8.2 ms/3.2 ms/12°, FOV = 25.6 cm, final resolution = 1 × 1x1mm) on a GE MR750 3 T scanner with an 8-channel headcoil. See supplementary material for additional information regarding group summaries for each scanning site.

### Quality control and attrition

All T1-weighted images were visually inspected to identify and exclude poor quality images. Following inspection, 17 images were excluded due to poor quality T1s, due to participant head movement (n = 16), and a neuroanatomical anomaly (n = 1). Additionally, two individuals had high quality T1 images, but were missing PCL-5 scores. The remaining 121 raw T1-images were retained and consisted of the final sample for data analyses.

### Image preprocessing

Preprocessing of the raw T1-weighted images was completed in FreeSurfer Version 6.0.0 (http://surfer.nmr.mgh.harvard.edu)^62^ on a Linux platform. FreeSurfer’s *reconall* command was used to preprocess all images through standard steps, including motion correction and averaging^63^, removal of non-brain tissue and skull-stripping^64^, automated Talariach transformation, segmentation of subcortical white and gray matter^62,63^, transformation to MNI305 atlas space, intensity normalization^65^, and volumetric registration. Technical details for FreeSurfer are described in detail elsewhere^62–64,66–72^.

### Image analysis

For all images, reconall continued without intervention through the processing stream following initial preprocessing steps. This included steps for another intensity normalization, white matter segmentation and editing, tessellation of white and gray matter boundaries and automated topology correction^73,68^, smoothing and surface inflation, spherical mapping and registration for each hemisphere^67,68,70^, and parcellation and labelling of cortical and subcortical structures. Cortical structures were labelled according to the Desikan-Killiany-Tourville parcellation (DKT)^74^ and mapped to the spherical surface, while subcortical and white matter structures are labelled according to FreeSurfer’s automated segmentation tool (aseg)^58^ and mapped to the MNI305 common space. Final statistics for GMV, CT, and surface area estimates of each region by hemisphere in both the DKT and subcortical aseg parcellations were generated.

### Vertex-wise cortical thickness analysis

Two separate vertex-wise CT analyses were conducted to: (1) examine group differences between control and PTSD groups; and (2) examine whether PTSD symptom severity scores for each PTSD symptom cluster are associated with CT in the PTSD group only. To conduct these vertex-wise analyses of potential differences in CT across the brain, we first generated cortical surface files for each subject. Subject surface files were registered to the average template (fsaverage), which is constructed from scans of 40 healthy adult subjects^75^ and smoothed with a 15 mm full width half-maximum kernel in order to compare differences in CT across subjects. Next, vertex-wise linear model tests across the cortical surface were conducted using either group (control vs PTSD) or PTSD symptom severity scores for PTSD symptom clusters (i.e., re-experiencing, active avoidance, negative alterations in cognition and mood, and hyperarousal) as the main predictor variables for each separate set of analyses (i.e., group [PTSD vs control group] analysis, and PTSD symptom cluster analysis). Additionally, given that the control group consisted of non-trauma exposed women, exploratory analyses (see supplementary material) were conducted examining trauma exposure (i.e., number of IPV-related direct assaults experienced) as a main predictor of interest (as opposed to group: control vs PTSD). All comparisons were conducted using AFNI’s 3dttest +  + function^76^, controlling for participant age, image acquisition site (UW or UAMS), and current diagnoses of major depression or anxiety disorders. To control for multiple comparisons, we used the AFNI slow_surf_clustsim.py function to run clustsim on the group-level fsaverage surface. We specified the uncorrected p threshold at 0.001 and ran 10,000 Monte Carlo simulations to generate the minimum surface cluster area unlikely to be detected due to chance (p > 0.05). Finally, cortical regions with thickness that significantly related to either group or PTSD symptom severity for each PTSD symptom cluster above the area threshold were extracted using AFNI SurfClust.

### Gray matter region of interest selection

Gray matter regions of interest (ROIs) included the hippocampus and amygdala of both the left and right hemispheres. ROIs were selected a priori based on previous research suggesting reduced volume in PTSD as a result of various trauma exposures^22–24^.

### Gray matter data analysis

Following processing in Freesurfer, gray matter ROIs from the DKT parcellation were entered into multiple linear regression models. Two sets of models were tested for each hemisphere for both ROIs (amygdala and hippocampus). Each model started off including covariates of non-interest (age, site, estimated total intercranial volume [eTIV]), before adding the main predictors of interest (i.e., group, PTSD cluster symptom severity scores, respectively for each set of models), followed by the inclusion of additional covariates (i.e., current diagnosis of anxiety disorder and major depressive disorder, coded as separate variables) that are prevalent within PTSD, and therefore may explain unique variance. The model examining group differences in GMV used dummy-coded contrasts with the control group as the reference group. Models were estimated using the fitlm function in Matlab. In order to avoid an inflated Type 1 error rate, we corrected for multiple comparisons by dividing the *p*-value of 0.05 by 4 (*p* = 0.0125). Lastly, given that the control group consisted of non-trauma exposed women, exploratory analyses (see supplementary material) were conducted examining trauma exposure (i.e., number of IPV-related direct assaults experienced) as a main predictor of interest (as opposed to group: control vs PTSD).

## Results

### Participant characteristics

See Table 1 for a complete description of participant characteristics. Independent samples t-tests indicated that groups did not significantly differ in age (*t*(119) = 0.91, *p* = 0.364) or eTIV (*t*(119) =  − 1.44, *p* = 0.151). The control group had significantly greater number of years of education compared to the PTSD group (*t*(119) = − 2.92, *p* = 0.004).

**Table 1:** Participant characteristics.

	Control (n = 22)	PTSD (n = 99)	
Age (years)	31.68 ± 7.92	33.48 ± 8.48	
Education (years)	16.73 ± 2.47*	15.00 ± 2.51	
*Race*			
#(%), Caucasian	17 (77.27)	72 (72.72)	
#(%), Black/African–American	3 (13.63)	18 (18.18)	
#(%), Hispanic/Latino	2 (9.09)	2 (2.02)	
#(%), Asian	0 (0)	1 (1.01)	
#(%), Native American	0 (0)	1 (1.01)	
#(%), other	0 (0)	5 (5.05)	
TIV (mm^3^ × 10^6^)	1.44 ± 0.11	1.40 ± 0.12	
PTSD symptom severity			
Re-experiencing	–	10.80 ± 4.75	
Avoidance	–	5.38 ± 2.03	
Negative alterations in cognition/mood	–	16.12 ± 6.10	
Hyperarousal/reactivity	–	12.15 ± 4.90	
Overall	–	44.57 ± 14.95	
Trauma exposure (# of direct assaults experienced)	–	5.75 ± 2.93	
Current major depressive disorder, # (%)	–	30 (30.30)	
Current anxiety disorder, # (%)	–	67 (67.67)	

Values listed as M ± SD unless otherwise noted. *indicates significant (*p* < .05) group difference. TIV = estimated total intracranial volume.

### Cortical thickness

#### Control versus PTSD

A diagnosis of PTSD did not significantly (*p* > 0.05 corrected for whole brain comparison) predict differences in CT across the entire cortical surface after controlling for age, scanner site, anxiety, and depression.

#### PTSD symptom clusters

**Figure 1:**
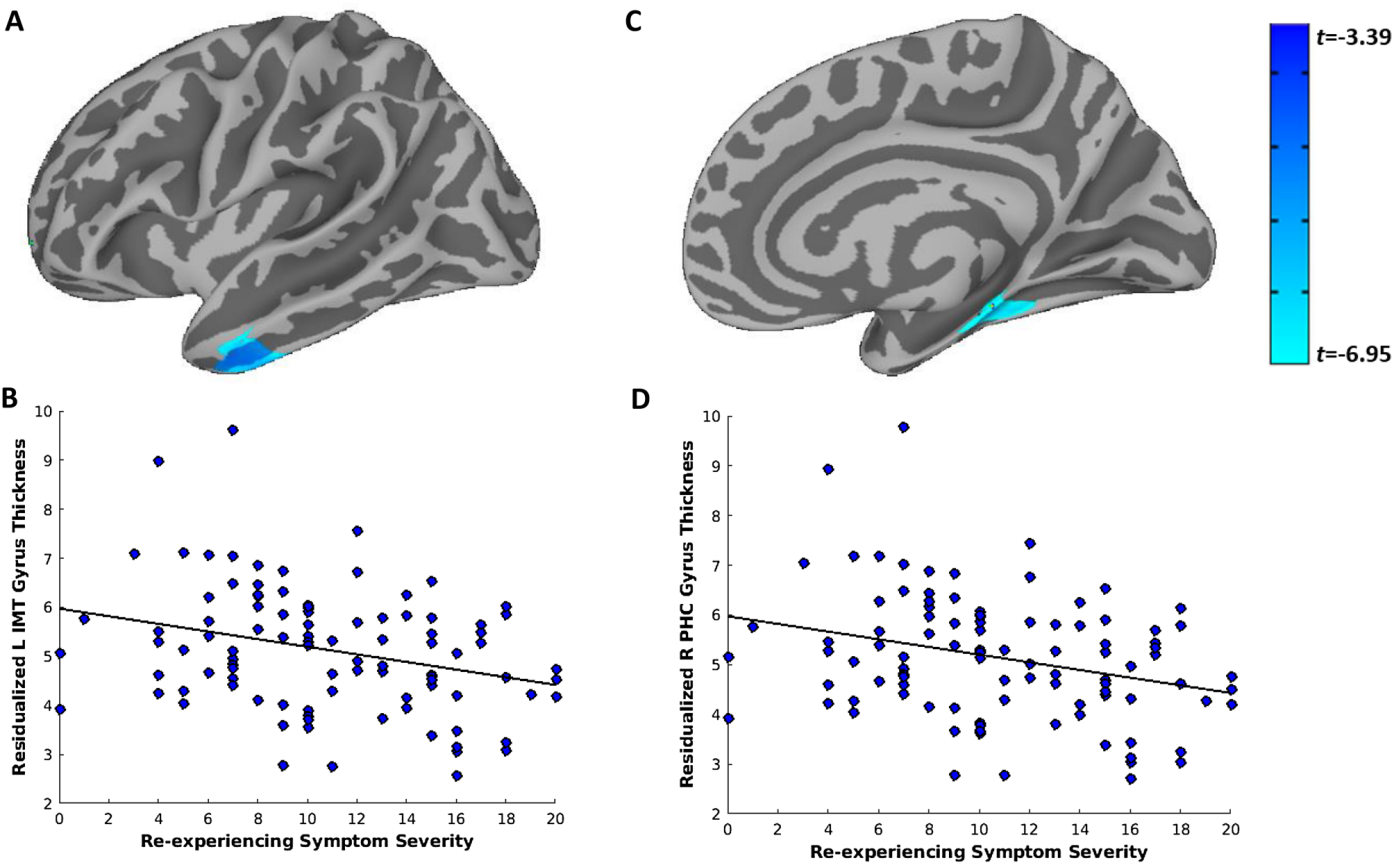
Greater Re-experiencing PTSD symptom severity is associated with decreased thickness of the left inferior and middle temporal gyrus and right parahippocampal gyrus. Vertex-wise linear model tests across the cortical surface revealed two clusters that survived Monte-Carlo correction for multiple comparisons and were associated with greater PTSD re-experiencing symptom severity: the left inferior and middle temporal gyrus (**A**,**B**) and the right parahippocampal gyrus (**C**,**D**). Re-experiencing symptom severity scores (cluster B; score range of 0–20) were obtained from the PTSD Checklist for DSM-V (PCL-5). PTSD = posttraumatic stress disorder; L = left; R = right; IMT = inferior/middle temporal; PHC = parahippocampal.

**Figure 2:**
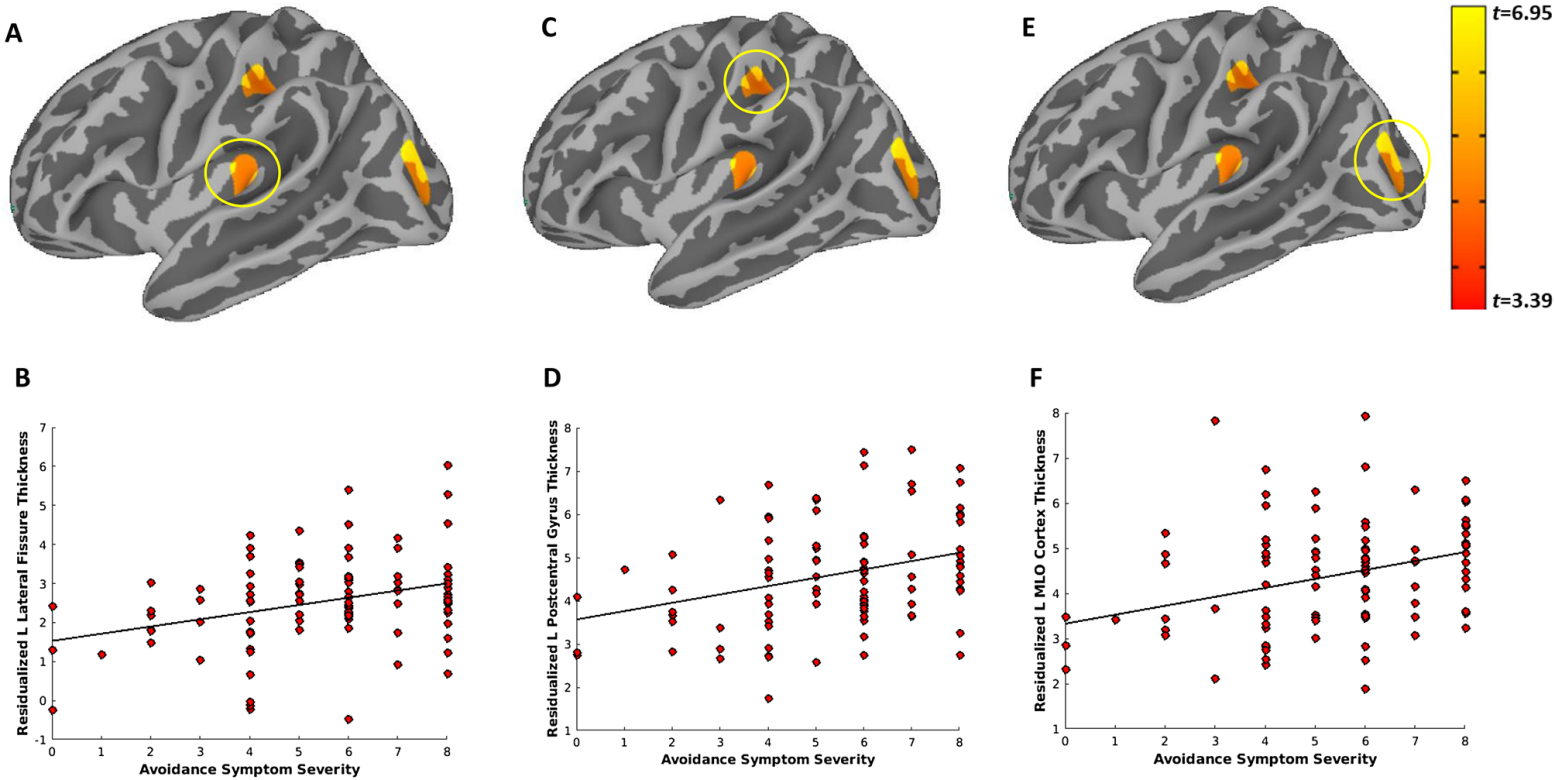
Greater Active Avoidance PTSD symptom severity is associated with increased thickness of the left lateral fissure, postcentral gyrus, and middle/lateral occipital cortex. Vertex-wise linear model tests across the cortical surface revealed three clusters that survived Monte-Carlo correction for multiple comparisons and were associated with greater PTSD re-avoidance symptom severity: the left lateral fissure (**A**,**B**), postcentral gyrus (**C**,**D**), middle/lateral occipital cortex (**E**,**F**). Active Avoidance symptom severity scores (cluster C; score range of 0–8) were obtained from the PTSD Checklist for DSM-V (PCL-5). PTSD = posttraumatic stress disorder. L = left; MLO = middle/lateral occipital.

**Figure 3:**
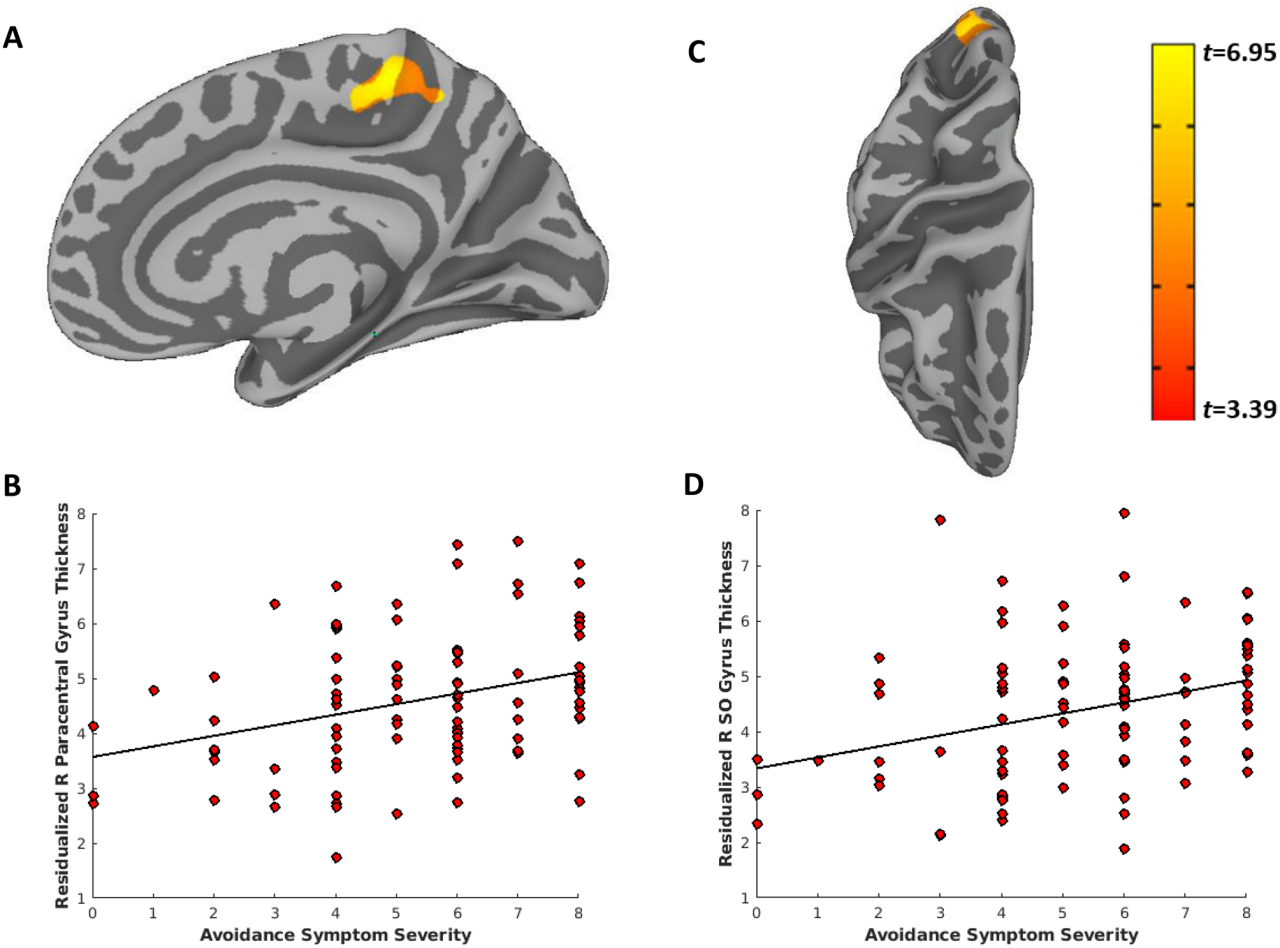
Greater Active Avoidance PTSD symptom severity is associated with increased thickness of the right paracentral, posterior cingulate, and superior occipital gyrus. Vertex-wise linear model tests across the cortical surface revealed two clusters that survived Monte-Carlo correction for multiple comparisons and were associated with greater PTSD re-experiencing symptom severity: the right paracentral and posterior cingulate (**A**,**B**), and superior occipital gyrus (**C**,**D**). Active Avoidance symptom severity scores (cluster C; score range of 0–8) were obtained from the PTSD Checklist for DSM-V (PCL-5). PTSD = posttraumatic stress disorder; R = right; SO = superior occipital.

Within the PTSD group, greater re-experiencing symptoms were significantly associated with decreased CT in the left inferior and middle temporal gyrus (Fig. 1A,B) and decreased CT in the right parahippocampal and medial temporal gyrus (Fig. 1C,D). Greater active avoidance symptoms were significantly associated with greater CT in the left lateral fissure (Fig. 2A,B), postcentral gyrus (Fig. 2C,D), and middle/lateral occipital gyrus (Fig. 2E,F) and greater CT in the right paracentral (Fig. 3A,B), posterior cingulate, and superior occipital gyrus (Fig. 3C,D). Symptoms of hyperarousal and negative alterations in cognition and mood did not significantly (*p* > 0.05 corrected for whole brain comparison) predict differences in CT across the entire cortical surface after controlling for age, scanner site, anxiety, and depression.

### Volumetric analyses

#### Control versus PTSD

**Figure 4:**
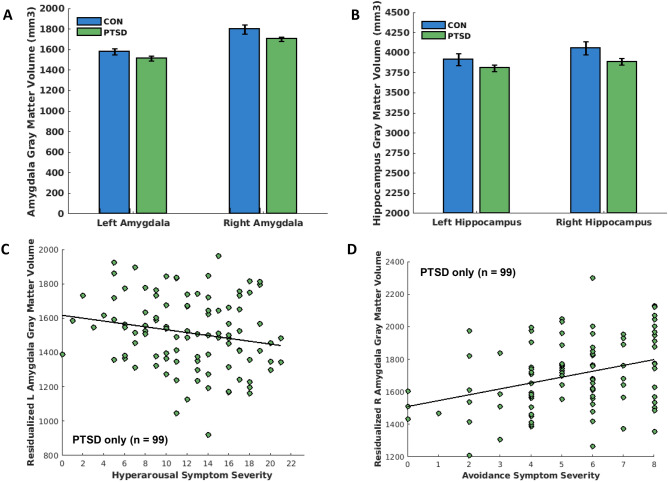
A diagnosis of PTSD in women does not predict differences in amygdala and hippocampal gray matter volume (**A**,**B**). However, within the PTSD group, greater hyperarousal symptoms significantly predicted less left amygdala volume, even after controlling for anxiety and depression (**C**); whereas greater active avoidance symptoms significantly predicted greater right amygdala volume, even after controlling for anxiety and depression (**D**). Hyperarousal (cluster E; score range of 0–24) and active avoidance (cluster C; score range of 0 to 8) symptom severity scores were obtained from the PTSD Checklist for DSM-V (PCL-5). PTSD = posttraumatic stress disorder; L = left; R = right.

A diagnosis of PTSD did not significantly predict differences in amygdala (left and right, *p*s = 0.739 and 0.636, respectively) or hippocampal (left and right, *p*s = 0.696 and 0.337, respectively) volume (see Table 2; Fig. 4A,B, respectively). There was a trend toward a current diagnosis of major depressive disorder predicting less right amygdala volume (*p* = 0.020; see Table 2).

**Table 2:** PTSD vs control group as predictor of hippocampus and amygdala gray matter volume.

	Step 1		Step 2		Step 3		Step 4	
Predictors	t-stat	P value	t-stat	P value	t-stat	P value	t-stat	P value
**Left amygdala**								
Age	− 1.02	.310	− 0.97	.334	− 1.07	.288	− 1.13	.259
Scanner site	− 0.38	.706	− 0.34	.731	− 0.38	.703	− 0.58	.560
TIV	8.39	< .001	8.31	< .001	8.31	< .001	8.10	< .001
Education	0.37	.713	0.23	.814	0.27	.788	0.06	.949
Group	–	–	− 0.48	.631	0.07	.947	0.33	.739
Anxiety	–	–	–	–	− 0.90	.368	− 0.75	.452
Depression	–	–	–	–	–	–	− 1.79	.076
R-squared	0.412		0.414		0.418		0.434	
Adjusted R-squared	0.392		0.388		0.387		0.399	
**Right amygdala**								
Age	− 1.32	.190	− 1.23	.221	− 1.21	.229	− 1.30	.196
Scanner site	− 2.45	.015	− 2.38	.018	− 2.36	.019	− 2.64	.009
TIV	7.15	< .001	7.06	< .001	7.03	< .001	6.81	< .001
Education	1.42	.158	1.15	.254	1.14	.257	0.89	.375
Group	–	–	− 0.91	.366	− 0.80	.423	− 0.47	.636
Anxiety	–	–	–	–	0.07	.944	0.26	.793
Depression	–	–	–	–	–	–	− 2.23	.027
R-squared	0.362		0.366		0.366		0.393	
Adjusted R-squared	0.340		0.339		0.333		0.355	
**Left hippocampus**								
Age	− 1.38	.167	− 1.39	.164	− 1.23	.219	− 1.27	.208
Scanner site	0.68	.492	0.67	.504	0.73	.466	0.61	.539
TIV	9.05	< .001	9.01	< .001	9.01	< .001	8.82	< .001
Education	1.52	.132	1.52	.132	1.47	.145	1.34	.181
Group	–	–	0.22	.829	− 0.54	.587	− 0.39	.696
Anxiety	–	–	–	–	1.39	.167	1.47	.144
Depression	–	–	–	–	–	–	− 0.98	.326
R-squared	0.484		0.484		0.493		0.497	
Adjusted R-squared	0.466		0.462		0.466		0.466	
**Right hippocampus**								
Age	− 1.43	.156	− 1.37	.172	− 1.21	.227	− 1.25	.214
Scanner site	0.55	.581	0.58	.561	0.64	.523	0.52	.606
TIV	8.88	< .001	8.80	< .001	8.80	< .001	8.60	< .001
Education	2.24	.026	2.04	.043	1.99	.048	1.86	.065
Group	–	–	− 0.50	.618	− 1.14	.258	− 0.96	.337
Anxiety	–	–	–	–	1.35	.179	1.44	.153
Depression	–	–	–	–	–	–	− 1.06	.290
R-squared	0.489		0.490		0.498		0.503	
Adjusted R-squared	0.471		0.468		0.472		0.472	

A diagnosis of PTSD did not significantly (*p* > .0125) predict differences in amygdala (left and right) and hippocampal (left and right) volume. TIV = total intracranial volume.

#### PTSD symptom clusters

Within the PTSD group, greater hyperarousal symptoms were significantly (*p* = 0.007) associated with less left amygdala volume, even after controlling for anxiety and depression (see Table 3; Fig. 4C). Greater active avoidance symptoms were significantly (*p* = 0.012) associated with greater right amygdala volume, even after controlling for depression and anxiety (see Table 3; Fig. 4D). Re-experiencing and negative alterations in cognition and mood symptoms did not significantly predict left (*p*s = 0.923 and 0.757, respectively) or right (*p*s = 0.424 and 0.803, respectively) amygdala volume. PTSD symptom cluster scores did not significantly predict left (*p*s = 0.134 to 0.647) or right (*p*s = 0.338 to 0.996) hippocampal volume (see Table 3).

**Table 3:** PTSD symptom clusters as predictors of amygdala and hippocampus grey matter volume.

	Step 1		Step 2		Step 3		Step 4	
Predictors	t-stat	P value	t-stat	P value	t-stat	P value	t-stat	P value
**Left amygdala**								
Age	− 0.97	.334	− 0.72	.474	− 0.82	.413	− 0.90	.369
Scanner site	− 1.10	.272	− 0.96	.340	− 0.99	.326	− 1.08	.281
TIV	8.55	< .001	7.55	< .001	7.53	< .001	7.41	< .001
Cluster B	–	–	− 0.08	.939	− 0.12	.907	0.10	.923
Cluster C	–	–	2.13	.035	2.19	.030	1.92	.058
Cluster D	–	–	0.07	.942	0.07	.946	0.31	.757
Cluster E	–	–	− 2.84	.005	− 2.73	.007	− 2.74	.007*
Anxiety	–	–	–	–	− 0.73	.464	− 0.67	.506
Depression	–	–	–	–	–	–	− 1.05	.298
R-squared	0.446		0.523		0.526		0.531	
Adjusted R-squared	0.429		0.486		0.483		0.484	
**Right amygdala**								
Age	− 0.92	.359	− 0.96	.342	− 0.93	.355	− 1.07	.287
Scanner site	− 2.53	.012	− 2.58	.011	− 2.56	.012	− 2.74	.007
TIV	7.39	< .001	6.11	< .001	6.07	< .001	5.97	< .001
Cluster B	–	–	− 1.17	.244	− 1.16	.249	− 0.80	.424
Cluster C	–	–	3.02	.003	2.98	.003	2.56	.012*
Cluster D	–	–	− 0.15	.878	− 0.15	.879	0.25	.803
Cluster E	–	–	− 1.46	.148	− 1.45	.151	− 1.47	.144
Anxiety	–	–	–	–	0.06	.951	0.17	.865
Depression	–	–	–	–	–	–	− 1.71	.089
R-squared	0.376		0.452		0.452		0.469	
Adjusted R-squared	0.357		0.409		0.403		0.415	
**Left hippocampus**								
Age	− 0.69	.491	− 0.58	.560	− 0.34	.736	− 0.42	.678
Scanner site	0.99	.322	1.22	.225	1.30	.196	1.20	.234
TIV	9.91	< .001	8.52	< .001	8.60	< .001	8.48	< .001
Cluster B	–	–	0.53	.598	0.62	.538	0.81	.421
Cluster C	–	–	1.05	.297	0.88	.381	0.64	.524
Cluster D	–	–	0.22	.828	0.23	.819	0.46	.647
Cluster E	–	–	− 1.32	.189	− 1.50	.136	− 1.51	.134
Anxiety	–	–	–	–	1.56	.121	1.63	.107
Depression	–	–	–	–	–	–	− 1.01	.314
R-squared	0.541		0.556		0.567		0.572	
Adjusted R-squared	0.527		0.521		0.529		0.529	
**Right hippocampus**								
Age	− 0.44	.659	− 0.39	.699	− 0.16	.874	− 0.26	.793
Scanner site	1.24	.219	1.52	.131	1.60	.113	1.47	.145
TIV	9.49	< .001	8.00	< .001	8.06	< .001	7.95	< .001
Cluster B	–	–	0.63	.533	0.71	.480	0.96	.338
Cluster C	–	–	1.03	.306	0.87	.386	0.57	.573
Cluster D	–	–	− 0.32	.751	− 0.31	.757	0.00	.996
Cluster E	–	–	− 0.48	.634	− 0.65	.521	− 0.65	.515
Anxiety	–	–	–	–	1.46	.149	1.54	.126
Depression	–	–	–	–	–	–	− 1.32	.189
R-squared	0.522		0.533		0.544		0.553	
Adjusted R-squared	0.507		0.497		0.503		0.508	

Within the PTSD group, greater hyperarousal symptoms significantly predicted less left amygdala volume, even after controlling for anxiety and depression (denoted *); while greater avoidance symptoms significantly predicted greater right amygdala volume, even after controlling for anxiety and depression (denoted *). PTSD symptom cluster scores within the PTSD group did not significantly (all *p*s > .0125) predict left or right hippocampal volume. TIV = total intracranial volume.

## Discussion

This study investigated CT and subcortical GMV among adult women with PTSD resulting from IPV-exposure and a group of healthy, age-matched women free of trauma exposure or mental health concerns. Results from the current study revealed that a diagnosis of PTSD among IPV-exposed women did not significantly predict differences in CT across the cortex or GMV in the amygdala or hippocampus compared to trauma-free women without PTSD. However, re-experiencing, active avoidance, and hyperarousal symptom severity within the PTSD group were significantly associated with differential (i.e., increased and decreased) CT in several regions and GMV in the amygdala.

Previous research among adults with PTSD, mostly male veterans, has consistently demonstrated reduced CT in several regions including the anterior cingulate cortex, prefrontal cortex (medial, ventral, dorsolateral), and inferior and superior temporal gyri^17,19,20,23,77^. Additionally, reductions in subcortical GMV within the hippocampus and amygdala compared to age-matched, healthy controls have also been consistently reported within this population^25,26^. Our findings, obtained from a vastly different population of women with PTSD following IPV-exposure are in contrast with these reports, as we found no differences in CT or GMV when group was dichotomized (PTSD vs control). Interestingly, our findings are consistent with a smaller (but more relevant) literature specifically focusing on homogenous samples of women with PTSD due to physical and sexual abuse. Consistent with our findings, Landre et al. examined a small sample of adult women with PTSD stemming from sexual abuse and found no differences in CT compared to controls^37^. Relatedly, there were no differences in hippocampal volume between women survivors of intimate partner violence and healthy controls^38^. Most recently, it was reported that adolescents with childhood sexual abuse exhibit preserved CT in the ventromedial prefrontal cortex, anterior cingulate gyri, middle temporal gyri, and superior temporal gyri compared to trauma-free controls^40^. Collectively, there is growing evidence suggesting that the specific type of trauma exposure (e.g., combat vs. IPV) may explain whether a diagnosis of PTSD predicts alterations in CT and GMV compared to healthy, trauma-free controls.

Although a diagnosis of PTSD did not predict differences compared to healthy controls, specific clusters of PTSD symptoms were associated with CT and GMV within women with IPV-related PTSD. These results suggest that altered brain structure within IPV-exposed women with PTSD may be driven by symptom severity within specific clusters (e.g., re-experiencing, avoidance, hyperarousal), or that these particular alterations led to an increased likelihood of the emergence of more severe symptoms. Moreover, our results revealed that the symptom clusters had a differential effect on brain structure. For instance, greater active avoidance symptoms were associated with greater CT in several regions and greater GMV in the right amygdala, whereas greater re-experiencing and hyperarousal symptoms were associated with decreased CT in several regions and decreased left amygdala volume. The observed differential relationships with the amygdala depending on PTSD symptom clusters is consistent with a recent meta-analysis that found among adults with PTSD, greater re-experiencing symptom severity scores were associated with greater left amygdala activity in response to threat, whereas greater avoidance symptom severity scores were associated with less amygdala activity in response to threat^78^. Moreover, a prior investigation reported a positive relationship between avoidance symptoms and right amygdala (and hippocampus) volume, and an inverse relationship between hyperarousal symptoms and left superior medial frontal gyrus volume in a small sample of adults (66% women) with non-combat related PTSD (87% of sample experienced IPV-exposure)^39^.

The differential relationships of PTSD symptom clusters with CT and GMV, in particular regions canonically implicated in PTSD (i.e., amygdala), highlights the importance of acknowledging that ‘PTSD’ is a diagnostic construct with considerable symptom heterogeneity^79^. Accordingly, researchers may be missing important structural correlates of PTSD in this specific population (women with IPV-exposure) and others (e.g., combat, motor vehicle accidents) by neglecting to examine the effect of each PTSD symptom cluster. For instance, our results revealed decreased CT in the parahippocampal gyrus of adults with greater re-experiencing symptoms. The parrahippocampal gyrus is a region of the cortex that surrounds the hippocampus and plays a role in encoding, and episodic, spatial, and contextual memory. Relatedly, re-experiencing symptoms involve memory retrieval processes. Although investigations are limited, there are two additional reports of decreased parahippocampal volume in PTSD populations (male combat veterans and natural disaster survivors) compared to controls^21,23^. Our findings add to a growing body of evidence suggesting that decreased CT in the parahippocampal gyrus may be a commonly observed structural alteration in PTSD, especially among those with greater re-experiencing symptoms. In contrast, greater avoidance symptoms were associated with greater CT in several regions (left lateral fissure and post central gyrus; right paracentral, posterior cingulate, and superior occipital cortex). Given that CT in these regions did not differ between controls and those with PTSD, further research is warranted to determine whether the observed structural alterations among women with PTSD with greater avoidance symptoms is clinically relevant. Lastly, it is worth noting that the addition of major depressive disorder (MDD) into our models yielded some intriguing patterns. For instance, there was an effect for depression to predict left and right amygdala volume in the between group analyses. Though it did not survive correction for multiple comparison, this may suggest that a current diagnosis of MDD, as opposed to PTSD, may be more predictive of amygdala volume in adult women with PTSD. Within the PTSD group, there was also a trend for depression to predict right amygdala volume. Although these findings were not statistically significant after controlling for multiple comparisons, and therefore their inclusion in the models did not influence the interpretation of the PTSD subgroup analyses, they highlight the potential importance of examining mental health comorbidities in addition to PTSD. For instance, Bremner et al. previously reported reduced hippocampal volume in women with PTSD from childhood sexual abuse and a comorbid diagnosis of MDD compared to individuals with current MDD without abuse, but only after controlling for PTSD^80^. While it is plausible that comorbid depression and anxiety may explain unique variance in predicting GMV, that does not appear to be the case for CT, as neither were associated with CT across the cortex in all tested models. As was done in the current study, it may be beneficial for future research investigations examining PTSD populations to present results with and without comorbid depression and anxiety disorder diagnoses included in statistical models in order to improve our understanding of each diagnoses unique contributions.

This study is notwithstanding limitations. For instance, the sample size of the control group (n = 22) was much smaller than that of the PTSD group (n = 99), which may potentially explain the lack of group differences in CT and GMV. However, the control group sample size is relatively greater than many of the previous neuroimaging studies that have yielded similar findings^36,37^, and the sample size of the PTSD group (n = 99) and overall sample (n = 121) affords greater statistical power than prior studies. Additionally, the control group consisted solely of non-trauma exposed adults, which conflates exposure to trauma with the manifestation of PTSD (for our PTSD group). However, exploratory analyses incorporating trauma exposure as a main predictor yielded similar findings as results obtained from including a dichotomous group classification (i.e., control vs. PTSD). Another potential limitation is that anxiety and depression were simply dichotomized based on whether participants had a current diagnosis (i.e., endorsed diagnostically significant symptoms). The inclusion of depressive and anxiety symptoms on a continuum may have explained more variance for those who were experiencing subthreshold symptoms. Additionally, this was a cross-sectional study, rendering it impossible to determine whether these structural alterations (or lack thereof) were present prior to, or a direct consequence of IPV-exposure and/or the development of PTSD. Moreover, over 80% of the sample was Caucasian. Although there were no significant differences in race between groups, this highlights the need for additional research with a larger and more racially diverse sample. Lastly, the current study did not track hormonal variations among all participants during the time of their scan, which is a limitation as recent various hormonal factors (e.g., birth control, menstrual cycle stage; sex hormones) are known to influence gray matter volume^81–84^. However, exploratory analyses among the PTSD group (for which data was available) revealed that results examining symptom severity within specific PTSD symptom clusters as a predictor of amygdala and hippocampal GMV did not differ based on whether birth control use was included as a covariate (see Supplementary Table 3).

In conclusion, the current study found no differences in CT or GMV in the amygdala or hippocampus of adult women with PTSD stemming from IPV-exposure, compared to trauma-free women without a diagnosis of PTSD. Although there were no group differences in the current study, re-experiencing, active avoidance, and hyperarousal symptom severity within the PTSD group were significantly associated with differential (increased and decreased depending on the region) CT in several regions and GMV in the amygdala. These findings add to the growing literature suggesting that: (1) the specific type of trauma exposure (e.g., interpersonal violence vs combat) may play an important role in determining whether a diagnosis of PTSD predicts structural brain differences compared to trauma-free controls, (2) alterations in CT and GMV among women with PTSD stemming from IPV-exposure may be driven by symptom severity within specific symptom clusters, as opposed to overall PTSD symptom severity, and (3) symptom severity within PTSD symptom clusters may have a differential effect on brain structure among women with IPV-related PTSD, with greater avoidance symptom severity predicting increased CT and GMV, and greater re-experiencing and hyperarousal symptom severity predicting decreased CT and GMV.

## Supplementary information

Supplementary Information.
