# Supplementary material for: Differential relationships of PTSD symptom clusters with cortical thickness and grey matter volumes among women with PTSD
Source: Sci Rep. 2021 Jan 19;11:1825. doi: 10.1038/s41598-020-80776-2 (PMC7815843; doi:10.1038/s41598-020-80776-2)
Supplement: Supplementary file 1 — Supplementary Information. [file 41598_2020_80776_MOESM1_ESM.docx]

**Supplementary Material: Differential relationships of PTSD symptom clusters with cortical thickness and grey matter volumes among women with PTSD**

Kevin M. Crombie^a^, Ph.D., Marisa C. Ross^a^, MPA, Allison M. Letkiewicz^a^, Ph.D., Anneliis Sartin-Tarm^a^, B.A., Josh M. Cisler^a^, Ph.D.

^a^Department of Psychiatry at the University of Wisconsin – Madison, 6001 Research Park Blvd, Madison, WI, USA 53719-1176

**Additional information on neuroimaging data acquisition for participants from PTSD and control groups:** Participants from the control and PTSD groups were scanned at both sites. Of the 121 participants included in the analyses, 64 participants (53% of overall sample) were scanned at site 1 (UAMS) and 57 participants (47% of overall sample) were scanned at site 2 (UW). Specifically, 11 participants from the control group (50% of control sample) and 53 participants from the PTSD group (53% of PTSD sample) were scanned at site 1; and 11 participants from the control group (50% of control sample) and 46 participants from the PTSD group (46% of PTSD sample) were scanned at site 2.

***Exploratory cortical thickness and volumetric analyses:*** Trauma exposure did not significantly (*p*>.001) predict differences in CT across the entire cortical surface after controlling for age, education, scanner site, anxiety, and depression. Additionally, trauma exposure did not significantly predict differences in amygdala (left and right, *p*s=.828 and .034, respectively) or hippocampal (left and right, *p*s=.187 and .079, respectively) volume (see Supplementary Table 1).

**Supplementary Table 1. Trauma exposure as predictor of hippocampus and amygdala gray matter volume**

| **Left Amygdala** | | | | | | | | |
| --- | --- | --- | --- | --- | --- | --- | --- | --- |
|  | Step 1 | | Step 2 | | Step 3 | | Step 4 | |
| Predictors | t-stat | P value | t-stat | P value | t-stat | P value | t-stat | P value |
| age | -1.02 | .310 | -0.86 | .389 | -0.94 | 0.348 | -1.04 | .302 |
| scanner site | -0.38 | .706 | -0.52 | .607 | -0.47 | .637 | -0.58 | .562 |
| TIV | 8.39 | <.001 | 8.37 | <.001 | 8.34 | <.001 | 8.09 | <.001 |
| education | 0.37 | .713 | 0.10 | .919 | 0.10 | .923 | -0.06 | .954 |
| trauma | - | - | -0.91 | .366 | -0.60 | .548 | -0.22 | .828 |
| anxiety | - | - | - | - | -0.77 | .345 | -0.58 | .560 |
| depression | - | - | - | - | - | - | -1.66 | .098 |
| R-squared | 0.412 | | 0.417 | | 0.420 | | 0.434 | |
| Adjusted R-squared | 0.392 | | 0.391 | | 0.389 | | 0.398 | |
| **Right Amygdala** | | | | | | | | |
|  | Step 1 | | Step 2 | | Step 3 | | Step 4 | |
| Predictors | t-stat | P value | t-stat | P value | t-stat | P value | t-stat | P value |
| age | -1.32 | .190 | -0.93 | .355 | -0.87 | .385 | -0.98 | .331 |
| scanner site | -2.45 | .015 | -2.88 | .004 | -2.89 | .004 | -3.03 | .002 |
| TIV | 7.15 | <.001 | 7.29 | <.001 | 7.27 | <.001 | 7.02 | <.001 |
| education | 1.42 | .158 | 0.67 | .501 | 0.68 | .501 | 0.51 | .611 |
| trauma | - | - | -2.59 | .010 | -2.59 | .010 | -2.13 | .034 |
| anxiety | - | - | - | - | 0.45 | .652 | 0.66 | .513 |
| depression | - | - | - | - | - | - | -1.84 | .068 |
| R-squared | 0.362 | | 0.397 | | 0.398 | | 0.415 | |
| Adjusted R-squared | 0.340 | | 0.371 | | 0.366 | | 0.379 | |
| **Left Hippocampus** | | | | | | | | |
|  | Step 1 | | Step 2 | | Step 3 | | Step 4 | |
| Predictors | t-stat | P value | t-stat | P value | t-stat | P value | t-stat | P value |
| age | -1.38 | .167 | -1.22 | .226 | -1.03 | .304 | -1.07 | .288 |
| scanner site | 0.68 | .492 | 0.52 | .604 | 0.43 | .667 | 0.38 | .703 |
| TIV | 9.05 | <.001 | 9.04 | <.001 | 9.14 | <.001 | 8.94 | <.001 |
| education | 1.52 | .132 | 1.18 | .241 | 1.20 | .233 | 1.12 | .264 |
| trauma | - | - | -1.01 | .315 | -1.53 | .127 | -1.33 | .187 |
| anxiety | - | - | - | - | 1.74 | .084 | 1.81 | .073 |
| depression | - | - | - | - | - | - | -0.74 | .461 |
| R-squared | 0.484 | | 0.488 | | 0.502 | | 0.504 | |
| Adjusted R-squared | 0.466 | | 0.466 | | 0.475 | | 0.473 | |
| **Right Hippocampus** | | | | | | | | |
|  | Step 1 | | Step 2 | | Step 3 | | Step 4 | |
| Predictors | t-stat | P value | t-stat | P value | t-stat | P value | t-stat | P value |
| age | -1.43 | .156 | -1.17 | .243 | -1.01 | .315 | -1.05 | .297 |
| scanner site | 0.55 | .581 | 0.30 | .766 | 0.22 | .827 | 0.17 | .867 |
| TIV | 8.88 | <.001 | 8.92 | <.001 | 8.99 | <.001 | 8.78 | <.001 |
| education | 2.24 | .026 | 1.73 | .086 | 1.74 | .083 | 1.66 | .099 |
| trauma | - | - | -1.59 | .114 | -2.01 | .047 | -1.77 | .079 |
| anxiety | - | - | - | - | 1.51 | .134 | 1.58 | .115 |
| depression | - | - | - | - | - | - | -0.80 | .423 |
| R-squared | 0.489 | | 0.500 | | 0.510 | | 0.512 | |
| Adjusted R-squared | 0.471 | | 0.470 | | 0.484 | | 0.482 | |

*Note.* The number of IPV-related direct assaults experienced (i.e., trauma exposure) did not significantly (*p* > .0125) predict differences in amygdala (left and right) and hippocampal (left and right) volume. TIV = total intracranial volume.

| **Supplementary Table 2. Correlation table of assessment factors** | | | | | | | | |
| --- | --- | --- | --- | --- | --- | --- | --- | --- |
| **Assessments** | **1** | **2** | **3** | **4** | **5** | **6** | **7** | **8** |
| **1. Overall PTSD symptom severity** (PCL-5) | - | - | - | - | - | - | - | - |
| **2. Re-experiencing symptoms** (PCL-5) | 0.842 | - | - | - | - | - | - | - |
| **3. Avoidance symptoms** (PCL-5) | 0.657 | 0.552 | - | - | - | - | - | - |
| **4. Negative alterations in cognition/mood symptoms** (PCL-5) | 0.866 | 0.563 | 0.478 | - | - | - | - | - |
| **5. Hyperarousal symptoms** (PCL-5) | 0.86 | 0.635 | 0.434 | 0.65 | - | - | - | - |
| **6. Current depression** (SCID-IV) | 0.323 | 0.321 | 0.016 | 0.334 | 0.255 | - | - | - |
| **7. Current anxiety** (SCID-IV) | 0.145 | 0.098 | 0.152 | 0.099 | 0.150 | 0.079 | - | - |
| **8. Trauma exposure** (NSA) | 0.197 | 0.27 | 0.093 | 0.107 | 0.150 | 0.215 | -0.015 | - |
| *Note*. Reported values are Pearson correlation coefficients. PCL-5 = PTSD Checklists for DSM-5; SCID-IV = Structured Clinical Interview for DSM-IV; NSA = National Women's Survey trauma assessment section. | | | | | | | | |
|  |  |  |  |  |  |  |  |  |

***Exploratory gray matter volume analyses examining birth control use as covariate***: As mentioned in the limitations section, the current study did not track hormonal variations among all participants during the time of their scan. However, exploratory analyses among the PTSD group (for which data was available for 83/99 participants) revealed that results examining PTSD symptom clusters as a predictor of amygdala and hippocampal GMV did not differ based on whether or not birth control use was included as a covariate (see Supplementary Table 3).

**Supplementary Table 3. Post-hoc exploratory analyses of PTSD symptom clusters as predictors of amygdala and hippocampus grey matter volume with and without birth control included as covariate**

| **Left Amygdala** | | | | | |
| --- | --- | --- | --- | --- | --- |
|  | Model 1- birth control use not included as covariate | | Model 2- birth control use included as covariate | |  |
| Predictors | t-stat | P value | t-stat | P value |  |
| age | -0.29 | .769 | -0.30 | .764 |  |
| scanner site | -0.20 | .840 | -0.15 | .882 |  |
| TIV | 5.67 | <.001 | 5.70 | <.001 |  |
| education | 0.04 | .968 | 0.00 | .999 |  |
| Cluster B | 0.53 | .594 | 0.69 | .493 |  |
| Cluster C | 2.24 | .028 | 2.31 | .023 |  |
| Cluster D | 0.14 | .889 | 0.32 | .747 |  |
| Cluster E | -2.51 | .014 | -2.69 | .009 |  |
| anxiety | -0.76 | .450 | -0.86 | .391 |  |
| depression | -1.34 | .183 | -1.47 | .147 |  |
| birth control | - | - | 1.32 | .190 |  |
| R-squared | 0.543 | | 0.554 | |  |
| Adjusted R-squared | 0.479 | | 0.484 | |  |
| **Right Amygdala** | | | | | |
|  | Model 1- birth control use not included as covariate | | Model 2- birth control use included as covariate | |  |
| Predictors | t-stat | P value | t-stat | P value |  |
| age | -0.44 | .665 | -0.44 | .658 |  |
| scanner site | -1.64 | .106 | 1.59 | .117 |  |
| TIV | 4.23 | <.001 | 4.27 | <.001 |  |
| education | 1.33 | .186 | 1.30 | .198 |  |
| Cluster B | 0.17 | .865 | 0.35 | .729 |  |
| Cluster C | 2.68 | .009 | 2.78 | .007 |  |
| Cluster D | -0.40 | .688 | -0.19 | .850 |  |
| Cluster E | -1.51 | .134 | -1.73 | .088 |  |
| anxiety | 0.03 | .977 | -0.09 | .930 |  |
| depression | -1.44 | .153 | -1.59 | .117 |  |
| birth control | - | - | 1.51 | .136 |  |
| R-squared | 0.486 | | 0.502 | |  |
| Adjusted R-squared | 0.414 | | 0.424 | |  |
| **Left Hippocampus** | | | | | |
|  | Model 1- birth control use not included as covariate | | Model 2- birth control use included as covariate | |  |
| Predictors | t-stat | P value | t-stat | P value |  |
| age | -0.32 | .745 | -0.33 | .746 |  |
| scanner site | 1.50 | .137 | 1.53 | .130 |  |
| TIV | 6.90 | <.001 | 6.88 | <.001 |  |
| education | 1.22 | .223 | 1.20 | .236 |  |
| Cluster B | 1.50 | .139 | 1.57 | .121 |  |
| Cluster C | 1.08 | .282 | 1.12 | .268 |  |
| Cluster D | 0.69 | .493 | 0.79 | .435 |  |
| Cluster E | -2.08 | .041 | -2.16 | .034 |  |
| anxiety | 1.28 | .206 | 1.21 | .230 |  |
| depression | -0.40 | .688 | -0.47 | .640 |  |
| birth control | - | - | 0.75 | .456 |  |
| R-squared | 0.611 | | 0.614 | |  |
| Adjusted R-squared | 0.557 | | 0.554 | |  |
| **Right Hippocampus** | | | | | |
|  | Model 1- birth control use not included as covariate | | Model 2- birth control use included as covariate | |  |
| Predictors | t-stat | P value | t-stat | P value |  |
| age | -0.23 | .822 | -0.23 | .819 |  |
| scanner site | 1.37 | .176 | 1.40 | .165 |  |
| TIV | 6.74 | <.001 | 6.73 | <.001 |  |
| education | 2.33 | .023 | 2.29 | .024 |  |
| Cluster B | 1.62 | .109 | 1.71 | .091 |  |
| Cluster C | 0.66 | .511 | 0.70 | .485 |  |
| Cluster D | 0.31 | .754 | 0.44 | .664 |  |
| Cluster E | -1.12 | .266 | -1.24 | .220 |  |
| anxiety | 1.10 | .274 | 1.03 | .308 |  |
| depression | -0.42 | .676 | -0.50 | .619 |  |
| birth control | - | - | 0.89 | .376 |  |
| R-squared | 0.616 | | 0.620 | |  |
| Adjusted R-squared | 0.562 | | 0.561 | |  |

Note. These results are from a sub-group of adults with PTSD (n=83) for which data pertaining to birth control use was available. Results were consistent across both models (i.e., model 1 – analyses with birth control use (yes/no) not included as a covariate; and model 2- analyses with birth control use (yes/no) included as a covariate.
